# All-trans retinoic acid and rapamycin normalize Hutchinson Gilford progeria fibroblast phenotype

**DOI:** 10.18632/oncotarget.4939

**Published:** 2015-08-13

**Authors:** Camilla Pellegrini, Marta Columbaro, Cristina Capanni, Maria Rosaria D'Apice, Carola Cavallo, Michela Murdocca, Giovanna Lattanzi, Stefano Squarzoni

**Affiliations:** ^1^ National Research Council of Italy, Institute of Molecular Genetics, IGM-CNR, 40136 Bologna, Italy; ^2^ Rizzoli Orthopaedic Institute, SC Laboratory of Musculoskeletal Cell Biology, 40136 Bologna, Italy; ^3^ Rizzoli Orthopaedic Institute, Laboratory RAMSES, 40136 Bologna, Italy; ^4^ Fondazione Policlinico Tor Vergata, U.O.C. Laboratory of Medical Genetics, 00133 Rome, Italy; ^5^ Tor Vergata University, Department of Biomedicine and Prevention, 00133 Rome, Italy

**Keywords:** Hutchinson Gilford progeria syndrome, premature aging, all-trans retinoic acid, rapamycin, DNA damage and repair

## Abstract

Hutchinson Gilford progeria syndrome is a fatal disorder characterized by accelerated aging, bone resorption and atherosclerosis, caused by a *LMNA* mutation which produces progerin, a mutant lamin A precursor. Progeria cells display progerin and prelamin A nuclear accumulation, altered histone methylation pattern, heterochromatin loss, increased DNA damage and cell cycle alterations. Since the *LMNA* promoter contains a retinoic acid responsive element, we investigated if all-trans retinoic acid administration could lower progerin levels in cultured fibroblasts. We also evaluated the effect of associating rapamycin, which induces autophagic degradation of progerin and prelamin A.

We demonstrate that all-trans retinoic acid acts synergistically with low-dosage rapamycin reducing progerin and prelamin A, via transcriptional downregulation associated with protein degradation, and increasing the lamin A to progerin ratio. These effects rescue cell dynamics and cellular proliferation through recovery of DNA damage response factor PARP1 and chromatin-associated nuclear envelope proteins LAP2α and BAF.

The combined all-trans retinoic acid-rapamycin treatment is dramatically efficient, highly reproducible, represents a promising new approach in Hutchinson-Gilford Progeria therapy and deserves investigation in ageing-associated disorders.

## INTRODUCTION

Lamin A/C, encoded by the *LMNA* gene, is a major component of the nuclear lamina, a three-dimensional matrix mostly layered at the internal surface of the inner nuclear membrane but present also in the nuclear interior. Lamin A/C is important for nuclear structure maintenance [1] and, in addition to its structural role, it is implicated in many key nuclear functions including chromatin organization, DNA replication, transcription, DNA repair and cell-cycle progression [2, 3]. Lamin A and C are alternative splicing products of the *LMNA* gene. While lamin C is synthesized directly, lamin A is first synthesized as a precursor (prelamin A) and then processed further by multiple proteolytic events at the carboxy-terminal end. Prelamin A is farnesylated at the C-terminus, a step that can be blocked by farnesyltransferase inhibitors (FTIs) and statins [4, 5], inhibiting all subsequent processing reactions. Then, a cleavage of the three C-terminal amino acids is operated by the specific zinc metalloprotease Ste24 (ZMPSTE24) [6, 7] and the C-terminal cysteine is methylated by the isoprenyl-cysteine carboxyl methyltransferase (ICMT). Subsequently, the maturing prelamin A undergoes a second cleavage, also mediated by ZMPSTE24, that removes the 15 C-terminal residues. This process results in mature lamin A.

Mutations in the *LMNA* gene give rise to a broad range of diseases collectively called laminopathies. Some of them, such as Hutchinson-Gilford Progeria Syndrome (HGPS), Mandibuloacral Dysplasia type A (MADA) and Familial Partial Lipodystrophy, Dunnigan type (FPLD2), are characterized by failure to process prelamin A, which accumulates within the nucleus thus producing aberrant chromatin structure [8].

HGPS (OMIM 176670) is a rare, dominant genetic disorder characterized by phenotypic features of accelerated aging, bone resorption (particularly at phalanges, clavicles and mandible), skin atrophy and atherosclerosis [9]. The disease appears in the first year of life and the children affected die at an average age of 13.5 years, predominantly from atherosclerosis and myocardial infarction or stroke [10, 11]. Most HGPS patients carry a de-novo point mutation (c.1824C > T; p.G608G) in heterozygosis [12–14]. This mutation creates a cryptic splice site within exon 11, which produces a mutant prelamin A form with an internal deletion of a 50 amino acids-long stretch containing the site for the last cleavage step. This shorter prelamin A, named progerin, is permanently farnesylated and carboxymethylated at its C-terminal domain and displays altered structure and biochemical properties with respect to prelamin A [4, 13, 15]. The most prominent HGPS cellular phenotypes include nuclear dysmorphism, heterochromatin alterations, chromosomal and telomere aberrations. Altered nuclear functions include compromised cell-cycle regulation, impaired DNA repair, increased apoptosis and senescence [11, 16, 17].

Currently, HGPS is an incurable disease. In the recent years, experimental studies were performed, *in vitro* and in animal models, with the goal of identifying novel targets for treating laminopathies and associated diseases [18, 19]. Different strategies were evaluated such as genetic approaches [20] and pharmacological treatments focused on modulation of prelamin A maturation process, in order to reduce the final amount of farnesylated progerin [21]. To date, two clinical trials have been performed, both relying on blocking the prelamin A maturation pathway [21, 22] through inhibition of the farnesylation step. Moreover, promising results have been obtained *in vitro* [23–25] by the use of rapamycin acting as an mTOR inhibitor which contributes to progerin degradation by activating autophagy [26].

Interestingly, a retinoic acid responsive element (L-RARE) has been identified within the *LMNA* promoter, and several studies suggest that all-trans retinoic acid (ATRA) may either enhance or reduce, depending on the cellular model, the promoter activity of different genes [27–31]. Retinoic acid was reported to enhance the *LMNA* promoter activity in murine P19 embryonal carcinoma cells [32, 33]; on the other hand, *LMNA* downregulation has been also described following ATRA treatment in human alveolar carcinoma and in HL-60 cells [28, 29].

Retinoic acid is enzymatically derived from vitamin A, it regulates cell development and regeneration, and is a component of serum [30]. In addition, all-trans-retinoic acid has been found to induce autophagy [34, 35], a mechanism already proved to be involved in farnesylated prelamin A removal [23, 25, 36].

Aiming to reduce the intranuclear progerin accumulation, we investigated the effect of ATRA prolonged administration to HGPS skin fibroblasts. Moreover, in order to maximize the efficacy of treatment, we evaluated the cellular response upon combined administration of ATRA and rapamycin at low dosage.

## RESULTS

### Reduction of progerin amount in HGPS fibroblasts

**Figure 1:**
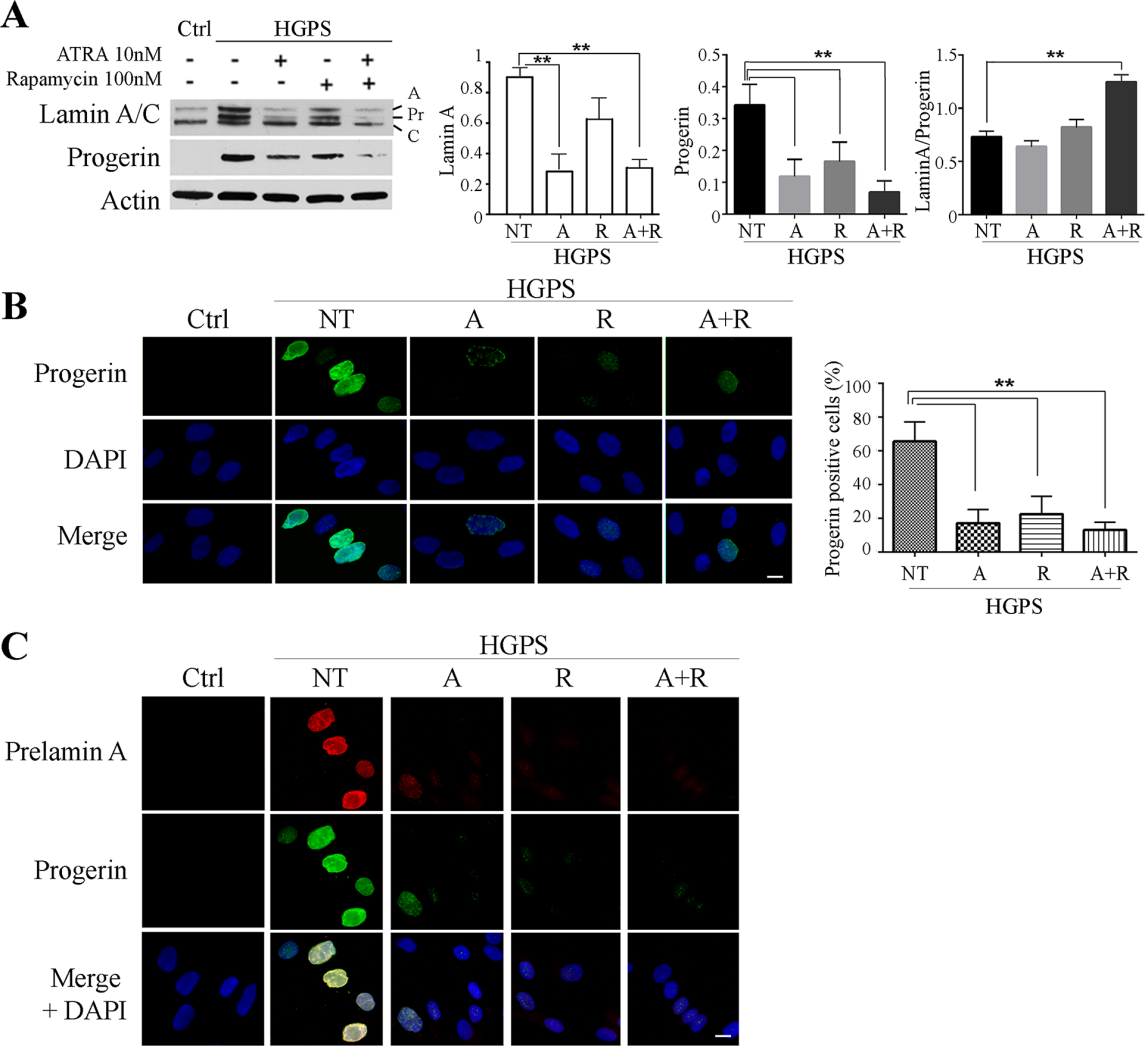
Progerin and prelamin A protein amount after 28 days of treatment **A.** Western blotting analysis of LaminA/C and progerin after 28 days of treatment. Actin was used as loading control. Relative densitometric analysis of lamin A, progerin and the ratio between themselves (Lamin A/Progerin) are reported on the right. ATRA, rapamycin and combined ATRA + rapamycin treatments are indicated as A, R and A+R respectively. **B.** Immunofluorescence evaluation of progerin (green) with nuclear staining (DAPI, blue). Histogram reports the progerin positive cells number. Ctrl: Healthy fibroblasts; NT: not treated HGPS fibroblasts; A, R and A+R: HGPS fibroblasts treated with ATRA, rapamycin and combined ATRA + rapamycin respectively. **C.** Double labelling of Prelamin A (red) and progerin (green); DAPI nuclear staining (blue). Ctrl: Healthy fibroblasts; NT: not treated HGPS fibroblasts; A, R and A+R: HGPS fibroblasts treated with ATRA, rapamycin and combined ATRA + rapamycin respectively. Data are presented as means ± SD (*n* = 3); *P* = 0.01(**); *P* = 0.05 (*). Scale bar, 10 μM.

ATRA treatment was able to reduce the amount of progerin in HGPS cells and resulted more effective than rapamycin treatment (Fig. 1A–1B). It should be noted that the rapamycin dosage used in this study is much lower than the one tested in previous studies [25, 36], which is expected to reduce any possible toxicity [24]. However, combined treatment with ATRA (10 nM) and rapamycin (100 nM) elicited the most significant reduction of progerin levels (Fig. 1A–1B). In a preliminary time-course experiment we monitored the effect of treatments up to four weeks. Using ATRA and rapamycin at the concentrations mentioned above, we observed a strong progerin decrease in comparison with untreated HGPS cells starting from day 14 (Fig. S1A, Fig. S2B) and optimal results were obtained at day 28. Thus, a comprehensive evaluation of different parameters was carried out at this time point and reported hereafter, except when differently specified.

Western blot analysis showed a decreased amount of both lamin A and progerin in all treated cells, while no appreciable variation of lamin C was visible (Fig. 1A); interestingly a significant increase of the relative amount of lamin A versus progerin was detected in ATRA plus rapamycin-treated cells, since the ratio between the two proteins raised from about 0.75 to about 1.3 (Fig. 1A). The western blot data were confirmed by immunofluorecence analysis with anti-progerin antibody and by quantitating the progerin positive cells (Fig. 1B, low magnification is shown in Fig. S1B).

We further evaluated the expression of wild-type prelamin A in HGPS cells by double immunofluorescence labeling in parallel with progerin. As expected, control nuclei were devoid of prelamin A, while untreated HGPS nuclei were strongly positive. The combined treatment with ATRA and rapamycin led to a remarkable drop in prelamin A positivity (Fig. 1C).

### Recovery of nuclear shape and chromatin organization in HGPS fibroblasts

**Figure 2:**
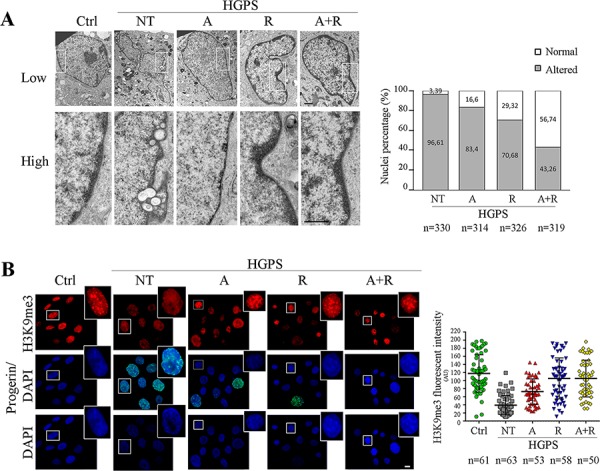
Analysis of nuclear shape and chromatin organization following 28 day of treatment **A.** Transmission electron microscopy analysis (TEM) of control and HGPS fibroblasts nuclei. High magnification details correspond to white gated area in the low magnification image of each sample. Lateral histogram displays the percentage of morphologically altered (grey) and normal (white) nuclei. The number of counted nuclei is indicated under the histogram (n). Ctrl: Healthy fibroblasts; NT: not treated HGPS fibroblasts; A, R and A+R: HGPS fibroblasts treated with ATRA, rapamycin and combined ATRA + rapamycin respectively. Scale bar, 1 μM. **B.** Co-labeling of tri-methylated histone H3 at Lysine 9 (H3K9me3) used to identify the heterochromatin arrangement (red), and progerin amount (green). Each single figure is a combination of three images onto a single figure. Dot-plot indentifies the mean intensity fluorescence of H3K9me3 calculated on nucleus area. The number of considered nuclei is indicated under the histogram (n). Ctrl: Healthy fibroblasts; NT: not treated HGPS fibroblasts; A, R and A+R: HGPS fibroblasts treated with ATRA, rapamycin and combined ATRA + rapamycin respectively. Scale bar, 10 μM.

Both ATRA and rapamycin had a positive effect on the recovery of nuclear shape and chromatin structure in HGPS fibroblasts. The number of cells with severe nuclear alterations appeared dramatically reduced by the single treatments, as demonstrated by transmission electron microscopy analysis (Fig. 2A). It is interesting to note that ATRA mainly contributed to restoring the roundish nuclear shape, while rapamycin was more effective in rescuing chromatin organization. In fact, as displayed in the high magnification details (Fig. 2A, lower panel) large scale chromatin organization was restored by rapamycin treatment and heterochromatin areas were clearly visible at the nuclear periphery, adherent to the nuclear lamina and spread in the nucleoplasm as small clusters. However, the best effect on nuclear organization was obtained by the combined (ATRA + rapamycin) treatment, which elicited both shape improvement and heterochromatin recovery (Fig. 2A). In order to better define the effects of treatments on chromatin arrangement we evaluated histone modifications. Histone 3 trimethylated on the lysine 9 residue (H3K9me3) is a marker of constitutive heterochromatin, reported to show an altered distribution pattern in HGPS nuclei [5, 37, 38]. Consistently, we found an abnormally uniform fluorescence over the nucleoplasm in untreated nuclei. The distribution pattern of H3K9me3 labeling was increasingly recovered by ATRA, rapamycin and combined ATRA plus rapamycin treatment in an increasing number of nuclei, which showed intranuclear fluorescent foci resembling those observed in controls (Fig. 2B).

Mean fluorescence intensity of H3K9me3 was lower than control values in untreated HGPS samples, but it was comparable to controls in samples subjected to rapamycin or combined ATRA plus rapamycin treatments (Fig. 2B, dot plot). We also assessed the amount of total H3 and the relative amounts of the monomethylated (H3K9me) and trimethylated (H3K9me3) fractions. In untreated HGPS cells, total H3 was decreased but the H3K9me3 fraction was proportionally elevated. The treatment restored an higher level of total H3 whose H3K9me3 fraction was proportionally reduced (Fig. S2A).

### Effects of treatments on Lamin A/C functional platforms

**Figure 3:**
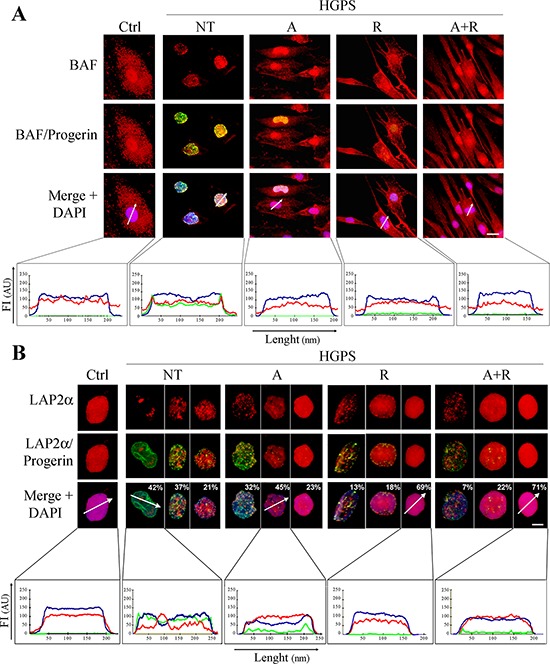
Analysis of chromatin and lamin-associated functional platforms after 28 days of treatment Immunofluorescence analysis of BAF **A.** or LAP2α **B.** (red) in co-labeling with progerin (green). The distribution pattern is described through the intensity profile histograms reported under panels A and B. White arrows indicate the X-axis of the histograms. The blue, red and green lines correspond to signals of DNA, BAF or LAP2α and progerin respectively. The percentages indicated in panel B report the relative amount of the different cell subpopulation present in each sample and displaying analogous labeling pattern. Intensity profiles were performed only for the nucleus corresponding to most represented subpopulation. Ctrl: Healthy fibroblasts; NT: not treated HGPS fibroblasts; A, R and A+R: HGPS fibroblasts treated with ATRA, rapamycin and combined ATRA + rapamycin respectively. Scale bars, 10 μM (A) and 5 μM (B), respectively.

Distribution of Barrier to Autointegration Factor (BAF), a bridging protein representing a key component of the lamin-DNA complex, was studied because of its known involvement in chromatin organization and its specific alteration in the presence of prelamin A or progerin [39]. For these reasons, BAF can be used as a marker for monitoring the nuclear functional integrity. In normal conditions, BAF is distributed in the cytoplasm and nucleus. Upon accumulation of any lamin A precursor, BAF is recruited to the nuclear lamina, regardless of the normal or mutated sequence of accumulated prelamin A. In untreated HGPS cells, BAF exhibited perfect co-localization with progerin at the nuclear rim (Fig. 3A). Normal BAF localization in nucleus and cytoplasm was restored upon drug treatments and the best distribution pattern was obtained in HGPS cells subjected to combined ATRA plus rapamycin administration (Fig. 3A). The analysis of the fluorescence intensity profiles confirmed an even nuclear and cytoplasmic distribution of BAF, the absence of BAF fluorescence peaks at the nuclear envelope and an extremely low progerin signal (Fig. 3A, lower panels).

Analogous results were obtained by analyzing the distribution pattern of Lamina-Associated Polypeptide 2α (LAP2α). LAP2α is known to associate with HMGN5 nucleosomal protein in the context of a protein platform regulating chromatin mobility and heterochromatin condensation [37]. In control human fibroblasts, LAP2α is localized in the nucleoplasm and interacts with nucleoplasmic lamin A [40]. In the presence of progerin, LAP2α is mislocalized to the nuclear periphery or redistributed in irregular foci in the nuclear interior (Fig. 3B). The treatments progressively restored a normal, evenly distributed fluorescence pattern in the nucleoplasm, again bearing the maximum efficacy when using both ATRA and rapamycin (Fig. 3B). Fluorescence intensity profile analysis (Fig. 3B, lower panels) further supported this data.

### Combined ATRA plus rapamycin treatment lowers DNA damage

**Figure 4:**
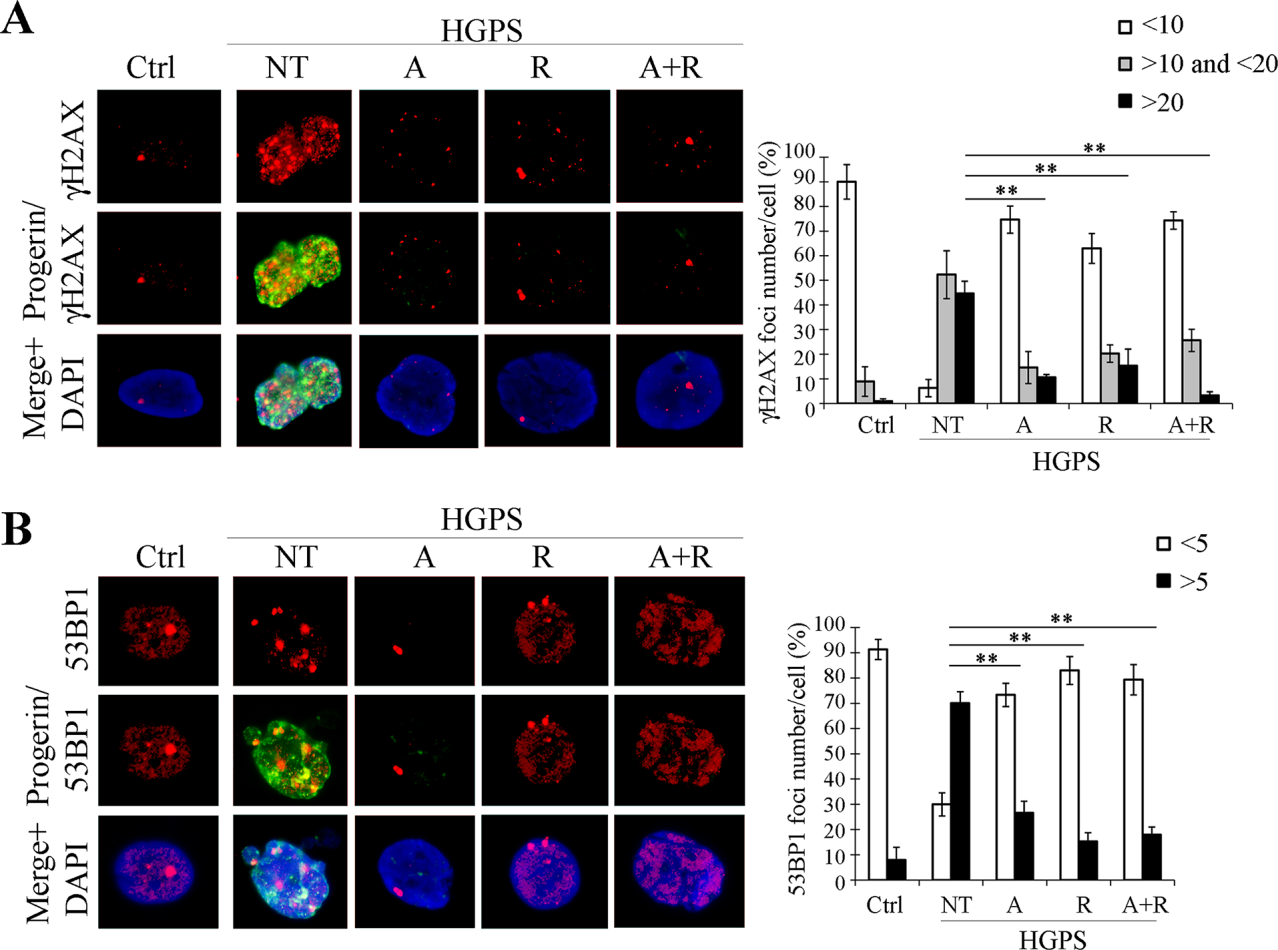
Analysis of DNA damage and repair markers after 28 days of treatment **A.** Double labeling with γH2AX (red) and progerin (green) with relative quantification of foci number per nucleus. **B.** Double labeling with 53BP1 (red) and progerin (green) with relative quantification of foci number per nucleus. Ctrl: Healthy fibroblasts; NT: not treated HGPS fibroblasts; A, R and A+R: HGPS fibroblasts treated with ATRA, rapamycin and combined ATRA + rapamycin respectively. Data are presented as means ± SD (*n* = 3); *P* = 0.01(**); *P* = 0.05 (*). Scale bar, 5 μM.

We also investigated levels and distribution of DNA damage response markers γH2AX and 53BP1. Both proteins were highly positive in HGPS nuclei, appearing as clusters within the nucleoplasm, indicating DNA damage in untreated cells (Fig. 4A and 4B). Treatment with ATRA or rapamycin considerably reduced the number of cells with high number of foci (>20 γH2AX foci/cell, > 5 53BP1 foci/cell) (Fig. 4A and 4B). Again, the highest efficiency was reached when the drugs were used in combination (Fig. 4A and 4B).

**Figure 5:**
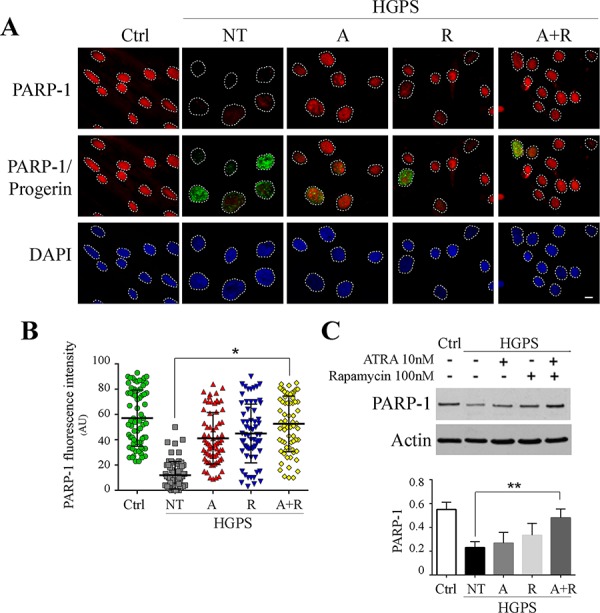
PARP-1 recovery following 28 days treatments administration **A.** Immunoflurescence analysis of PARP-1 (red) co-labeled with progerin (green) and DAPI staining (blue). Each single figure is a combination of three images onto a single figure. **B.** Analysis of fluorescence intensity mean of PARP-1 for each experimental conditions calculated on nucleus area. **C.** Western blotting of PARP-1 and relative quantification. Actin was used as loading control. Ctrl: Healthy fibroblasts; NT: not treated HGPS fibroblasts; A, R and A+R: HGPS fibroblasts treated with ATRA, rapamycin and combined ATRA + rapamycin respectively. Data are presented as means ± SD, *n* indicates the number of analyzed nuclei. *P* = 0.01(**); *P* = 0.05 (*). Scale bar, 10 μM.

In addition, we investigated the expression of poly(ADP-ribose) polymerase 1 (PARP1), a chromatin-associated enzyme involved in the regulation of various important cellular processes and also in the choice between activation of homologous recombination (HR) or non-homologous end joining (NHEJ) DNA repair mechanism. It is reported that the accumulation of progerin causes PARP1 down-regulation and, by interfering with PARP1 nuclear import, leads to proliferation defects (prolonged mitosis) and error-prone DNA repair via NHEJ [41]. Accordingly, immunofluorescence analysis showed a low amount of PARP1 in HGPS nuclei in comparison with controls (Fig. 5A). Treatment with ATRA or rapamycin caused an increase in fluorescence positivity, which reached the highest level using both drugs in combination (Fig. 5A). Evaluation of mean fluorescence intensity confirmed this data, showing a progressive amelioration with ATRA, rapamycin and ATRA plus rapamycin, respectively, finally showing a PARP1 staining pattern comparable to control fibroblasts (Fig. 5B). We further evaluated PARP1 protein expression by western blot analysis. We could clearly observe an increasing trend of the PARP1 amount caused, respectively, by treatment with ATRA, rapamycin and both drugs, confirming the immunofluorescence analysis results.

### Improvement of cell proliferation and reduction of G2/M cell population

**Figure 6:**
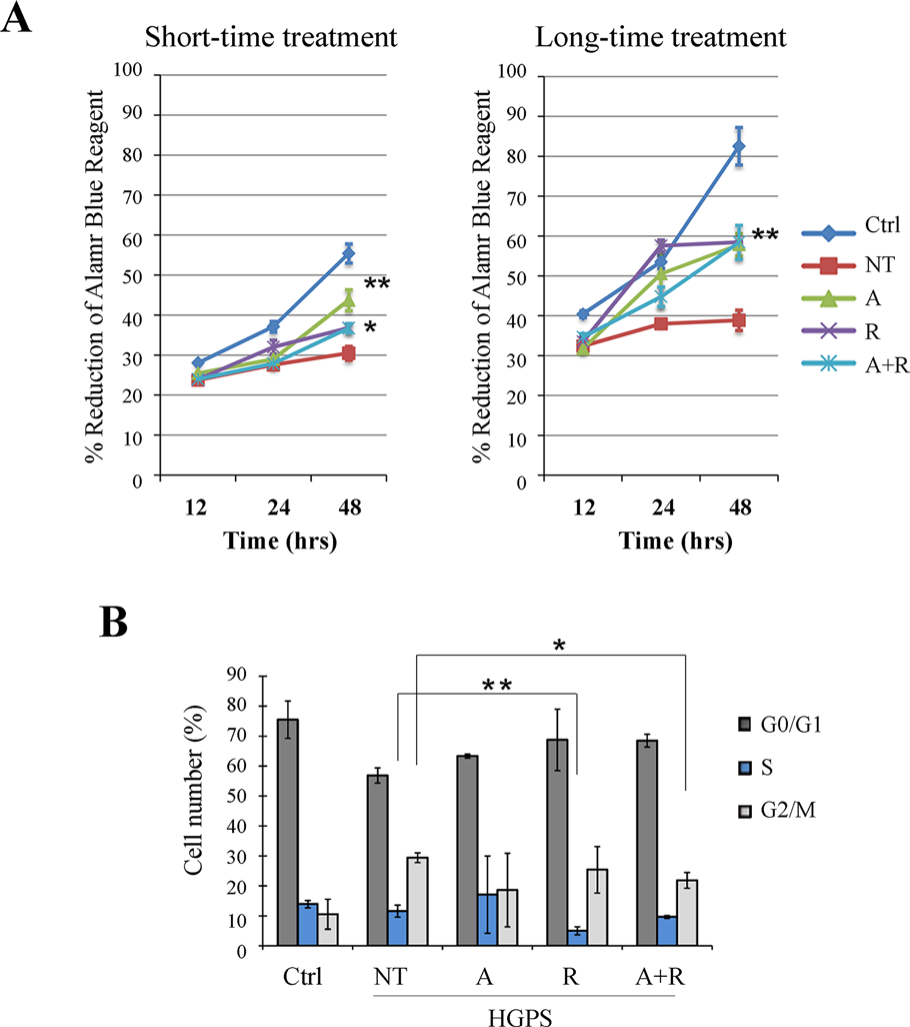
Evaluation of treatment effects on cellular proliferation ability and cell cycle **A.** Alamar Blue proliferation assay performed at short-time treatment (48 hrs) and at long-time treatment (48 hrs of monitoring, carried out after 28 days of treatment). After the first 12 hrs of adhesion the cells were monitored for colorant reduction at 24 and 48 hrs maintaining the samples under treatment. **B.** FACS analysis of cell cycle after 28 days of treatments. Data are presented as means ± SD (*n* = 3) and treated samples (A, R, A+R) are compared with not treated sample (NT). *P* = 0.01(**); *P* = 0.05 (*). Ctrl: Healthy fibroblasts; NT: not treated HGPS fibroblasts; A, R and A+R: HGPS fibroblasts treated with ATRA, rapamycin and combined ATRA + rapamycin respectively.

The cell proliferation ability was monitored with Alamar Blue assay for 48 hrs in the presence or absence of treatments and in comparison to control fibroblasts. We performed the study in two conditions: short-time (after 48 hrs of treatment) and long-time (after 28 days of treatment) (Fig. 6A)

When compared to control cells, untreated HGPS cells showed a proliferation defect that was more evident after several population doublings, as shown in Fig. 6A (both left and right panels).

HGPS fibroblasts treated for 48 hrs (Fig. 6A, short-time treatment) with rapamycin and ATRA plus rapamycin demonstrated a slight improvement of cellular proliferation, while the most significant improvement was obtained by ATRA administration. Otherwise, treatment of HGPS cells for 28 days (Fig. 6A, long-time treatment), elicited a significant enhancement of growth rate in all experimental conditions.

Potential effects on the cell cycle were evaluated by cytofluorimetric analysis (Fig. 6B). In comparison with control cells, untreated HGPS cells showed an increased number of cells in G2/M phase, while S phase was slightly more populated. The treatment, specifically when combining ATRA with rapamycin, lowered the G2/M population and the frequency of S phase was more similar to controls (Fig. 6B).

### ATRA effects on *LMNA* gene expression

**Figure 7:**
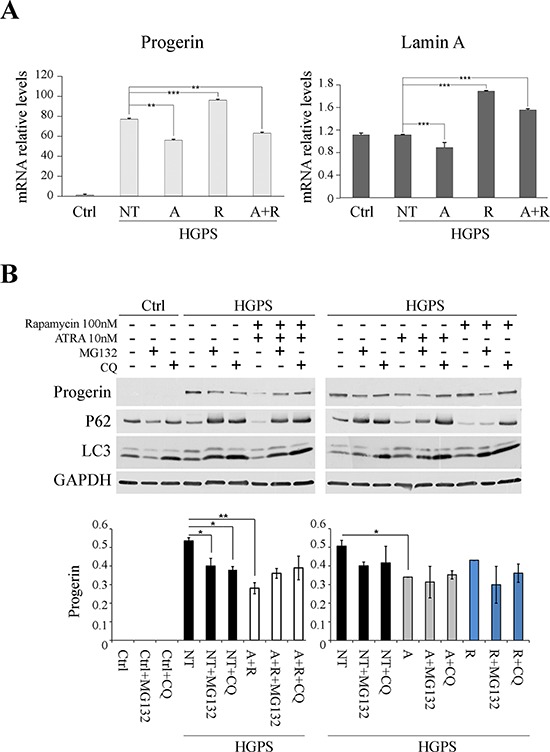
Effect of treatments on LMNA gene expression and on involvement of protein degradation pathways as the putative mechanisms of progerin reduction **A.** On the left: real-time qPCR of progerin in HGPS fibroblasts untreated (NT) and treated with ATRA (A), Rapamycin (R) and ATRA + Rapamycin (A+R), using CTRL sample as reference. The data were normalized to internal control GAPDH mRNA and are representative of three independent biological replicates. Values represent mean ± SEM. **p < 0.01, ***p < 0.001. On the right: real-time qPCR of lamin A in HGPS fibroblasts untreated (NT) and treated with ATRA (A), Rapamycin (R) and ATRA + Rapamycin (A+R), using CTRL sample as reference. The data were normalized to internal control GAPDH mRNA and are representative of three independent biological replicates. Values represent mean ± SEM. ***p < 0.001. **B.** Experiment of blocking either proteasome and autophagy protein degradation pathways. Samples, treated for 3 days with ATRA + Rapamycin (left panel) and ATRA or Rapamycin (right panel), were subject to MG132 and Chloroquine for 12 hrs before raising the cells. Analysis of progerin protein levels and evaluation of autophagy markers, P62 and LC3. GAPDH was used as loading control. Data are presented as means ± SD; *P* = 0.01(**); *P* = 0.05 (*).

RT-PCR analysis of progerin and *LMNA* mRNAs was performed in HGPS before and after ATRA, rapamycin and drug combination treatments. After treatment with ATRA, progerin transcript was dowregulated in comparison with untreated HGPS cells. In contrast, progerin mRNA expression was increased by rapamycin treatment, compared to untreated fibroblasts, demonstrating a strong positive feedback response, yet inefficient at the protein level due to the high autophagic degradation rate. The ATRA effect of mRNA downregulation was confirmed by observing progerin transcript in HGPS cells treated with a combination of both drugs: ATRA presence in rapamycin-treated fibroblasts brought back mRNA expression levels to values lower than those of untreated fibroblasts. An overlapping trend was observed for lamin A mRNA expression, indicating that progerin and lamin A transcript amounts were down-regulated by ATRA administration. (Fig. 7A).

### Involvement of protein degradation pathways in progerin decrease

In order to define the possible synergy of ATRA and rapamycin on progerin degradation, we evaluated the effect of these drugs on both autophagy, known to be involved in farnesylated prelamin A degradation, and ubiquitin (Ub)–proteasome pathway (UPP) (Fig. 7B).

We treated HGPS fibroblasts with rapamycin and ATRA, in combination (Fig. 7B, left panel) and singularly (Fig. 7B, right panel), for 72 hours, refreshing the treatments every day. The two pathways were blocked by the use of chloroquine (CQ) and MG132, respectively, added 12 hours before stopping the experiment. CQ impairs autophagy as it inhibits both fusion of autophagosomes with lysosomes and lysosomal protein degradation, while MG132 is a proteasome inhibitor, which reduces the degradation of ubiquitin-conjugated proteins. P62 and LC3 were labeled in these experiments to trace block of autophagic activity (Fig. 7B).

In HGPS cells co-treated with ATRA and rapamycin (Fig. 7B, left panel) we could observe progerin variations after both CQ and MG132 administration: after autophagy block, the progerin levels were raised to the same levels of untreated HGPS samples; a partial increase was observed also with UPP chemical blocking.

HGPS cells not treated with ATRA or rapamycin, but subjected to MG132 or CQ treatments, presented a decrease in progerin levels (Fig. 7B).

In HGPS cells treated with ATRA (Fig. 7B, right panel), CQ addition induced a restoration of progerin level which was comparable with the untreated HGPS samples. On the contrary, when MG132 was added to ATRA-treated samples, progerin was decreased because in this condition progerin removal via autophagy is enhanced [42].

In HGPS cells treated with rapamycin (Fig. 7B, right panel) the progerin level was unchanged with respect to untreated samples. This effect is due to the rapamycin concentration applied here (100 nM) which is lower with respect to concentrations reported to be effective in reducing progerin amount in previous studies [36]. Here, addition of either MG132 or CQ induced decrease or increase of progerin level, respectively, confirming involvement of rapamycin-dependent autophagic mechanism in progerin degradation [25].

These results demonstrated that the combined action of ATRA and rapamycin leads not only to downregulation of progerin expression, as stated in the previous paragraph, but also to protein degradation through the lysosomal and proteasomal pathways.

## DISCUSSION

### Lowering progerin levels

The results we show clearly indicate that ATRA is effective in lowering the amount of progerin in cultured skin fibroblasts derived from HGPS patients. Reduction of progerin amount was accompanied by recovery of many important nuclear markers referred to nuclear shape, chromatin organization, methylated histone distribution, DNA damage repair, cell cycling and proliferation.

In order to maximize progerin reduction, in view of a possible therapeutic application, we associated to ATRA a low dose of rapamycin because of its demonstrated efficacy in promoting progerin clearance by means of autophagic mechanism. Noteworthy, despite both lamin A and progerin amount decreased during ATRA and ATRA-rapamycin administration, the ratio between lamin A and progerin was shifted toward higher values by combining the two drugs. Also, decrease in prelamin A level was always detected in association with progerin decrease. This result represents an important issue because of the known toxicity of this lamin A precursor, e.g. in prelamin-accumulating laminopathies such as MADA and Restrictive Dermopathy. Importantly, the combined use of ATRA and rapamycin allowed us to employ rapamycin at much lower dosage with respect to those previously reported, which reduces drug toxicity and is particularly relevant in view of the design of therapeutic applications.

As a first conclusion we can remark the view that HGPS patients suffer from a double trouble consisting in accumulation of progerin first, and then of prelamin A [39, 43] and the treatment we describe here is efficient in recovering from both alterations.

### Cellular phenotype recovery

Together with the reduction in progerin amount we describe the recovery of the general nuclear morphology following treatment, including normal heterochromatin pattern restoration.

The ultrastructural morphology analysis of ATRA and rapamycin treated samples reveals regular nuclear profiles and a normal distribution of euchromatin and heterochromatin areas. In particular, ATRA contributes to restore the regular nuclear shape in HGPS cells, while rapamycin appears effective in the recovery of an organization based on distinguished heterochromatin and euchromatin domains. The combined treatment we tested improves both aspects of the nuclear structure. This effect is related to the reversal of the lamin A to progerin ratio we observe following the combined treatment.

Using immunofluorescence, we could detect an abnormal distribution pattern of H3K9me3, a marker of constitutive heterochromatin, in most HGPS nuclei, namely in those with higher progerin expression, featuring a diffuse H3K9me3 labelling; following treatment, a normalization of the organization was clearly detectable in an increasing number of nuclei, where H3K9me3 distributed as aggregates on the nuclear rim and at intranuclear clusters, according to published data [5, 37, 40].

In this regard, it has been reported that progerin as well as prelamin A sustain H3K9me3 anomalous increase by way of enhanced SUV39H1 binding to the lamin precursors, which prolongs the methylating activity of this enzyme. This causes defective DNA accessibility for the repair enzymes, early senescence and premature aging in Zmpste24−/− mice, as well as chromatin repression [44]. On the other hand, H3K9me3 has also been reported to be quantitatively decreased in HGPS [45] and other reports in the literature describe a decrease in the amount of trimethylated H3K9 on the basis of immunofluorescence data. The fluorescence reorganization we show for the H3K9me3 from a uniform, low-intensity, broadly distributed signal in abnormally large nuclei to condensed, high-intensity signal, localized in foci within smaller nuclei, may mimic an increase in amount. The western blot analysis, however, confirmed that total H3 is decreased in HGPS nuclei but the H3K9me3 fraction it contains is proportionally elevated. Treating the cells with the drugs restores an higher level of total H3 whose H3K9me3 fraction is proportionally reduced (Fig. S2A). It appears therefore that the normalization of H3K9me3 distribution we obtain is related to the decrease in progerin amount and/or to higher values of the lamin A/progerin ratio. Beside this, ATRA has been also reported to regulate chromatin acetylation by inhibiting HDACs and enhancing acetyltransferase (HAT) activity [46].

Thus it appears that in progeric nuclei there is a general chromatin functional impairment, maintained by the high amounts of progerin and farnesylated prelamin A, but following treatments a chromatin reorganization is attainable, which is more similar to the normal distribution into heterochromatin and euchromatin domains. In this regard it is worth to note that trichostatin A, an HDACs inhibitor, is also able to reduce progerin level to some extent [5]. Moreover, we previously demonstrated that the dispersed chromatin commonly observed in progeric nucleoplasm by transmission electron microscopy is not transcriptionally active as euchromatin normally is [5], but it remains in a particular “silenced but not compact” state that does not imply an higher affinity for the electron-dense staining media used for routine electron microscopy analysis, thus appearing electron-transparent, like euchromatin is reported to be.

PARP1 is involved in triggering DNA repair mechanism, cooperating in the choice between homologous recombination and non homologous end joining (NHEJ). In the absence or reduction of PARP1, DNA repair is shifted to NHEJ, which is a rapid error-prone DNA repair mechanism. In HGPS vascular smooth muscle cells, this imbalance leads to alteration of cell cycle with a prolonged G2/M phase due to an impairment of mitosis [47]. It has been demonstrated that PARP1 decreases with increasing level of progerin, a common event in cultured progeria cells, associated with increasing passage number [47]. Here we show that PARP1 is considerably reduced in HGPS fibroblasts, but treatment with ATRA and rapamycin is able to increase and restore PARP1 level, thus improving the DNA repair ability of these cells. Accordingly, we detect both improvement of γH2Ax and 53BP1 labeling pattern and a recovery of cytofluorimetric profiles showing decrease of G2/M phase in comparison with untreated HGPS cells.

Interestingly, PARP1 is also reported to associate with BAF and lamin A to modulate transcription and nucleosome organization [48]. Thus, recovery of BAF distribution [49] in ATRA plus rapamycin-treated cells may act synergistically with PARP1 recovery, leading to functional improvement of chromatin in HGPS cells. Moreover, combined ATRA plus rapamycin treatment is also able to restore a normal distribution of the bridging protein LAP2α throughout the nuclear interior, which further contributes to recovery of the BAF-containing protein platform, where LAP2α is a major partner [37].

### Effects on cell cycle

HGPS fibroblasts are characterized by senescence and, especially at high culture passages, display a duplication slowdown. The data we obtained through the cell population doubling assay demonstrate that ATRA and rapamycin are able to improve the cell proliferation rate. In comparison with controls, untreated HGPS fibroblasts displayed an increased population in G2/M phase probably related to accumulation of unrepaired DNA damage [50] and prolonged mitosis, a decreased G1 phase, while S phase was only slightly more populated. This result was expected, as already described in the literature [47] and reported in other progeroid laminopathies [37]. The treatment with ATRA and particularly when combining ATRA with rapamycin, reduced the G2/M population. Both ATRA and rapamycin, as well as their combination, contributed to slightly increase the frequency of cells in G1 phase, while the S phase population was only minimally affected by the treatment.

## MATERIALS AND METHODS

### Cell cultures

All cell samples were obtained following an informed consent by patients or families. The protocol was approved by the local ethical committee.

HGPS skin fibroblasts were obtained from two patients carrying the G608G LMNA mutation. Control skin fibroblast cultures were obtained from skin biopsies of two healthy patients (mean age 12) undergoing orthopaedic surgery. Cell cultures were established and grown in Dulbecco's modified Eagle's medium (DMEM) supplemented with 10% fetal calf serum (Gibco) and 1% penicillin/streptomycin. The experiments were performed between passages 13 and 20.

### Drug treatments

All experiments were carried out on proliferating cells and drug administration was performed as reported below. Rapamycin (0,1 μM, Sigma, Milano, Italy) and ATRA (10 nM, Sigma, Milano, Italy) were given to cultured fibroblasts for 7–28 days changing the drug-containing medium two times a week.

To block lysosomal activity (autophagy), chloroquine (Sigma, 25 μM) was applied for 12 hrs either in the presence or in the absence of rapamycin or ATRA. To check the ubiquitin (Ub)–proteasome pathway (UPP), proteasome inhibitor MG132 (Sigma, 1 μM) was applied for 12 hrs.

### Antibodies

Antibodies employed for Western blot analysis or immunofluorescence labeling were: Anti-Progerin, mouse monoclonal (ALX-804-662, Enzo Life Sciences); anti-lamin A/C, goat polyclonal (SC-6215, Santa Cruz Biotechnology); anti-prelamin A, goat polyclonal (SC-6214 Santa Cruz Biotechnology); anti-prelamin A, rabbit polyclonal (1188-2, Diatheva), raised against the last 15 aminoacids of the prelamin A sequence including the farnesylated cysteine residue but not the SIM sequence; anti-H3K9me3, mouse monoclonal (AB6001, Abcam) and rabbit polyclonal (07-442, Millipore); anti-monomethyl-H3K9, rabbit polyclonal (Abcam); anti-Histone H3, goat polyclonal (Santa Cruz Biotechnology C-16); anti-BAF, rabbit polyclonal (FL-89, Santa Cruz Biotechnology); anti-LAP2α, rabbit polyclonal (kind gift of Roland Foisner); anti-γH2AX, rabbit polyclonal 4937 Cell Signalling); anti-53BP1, rabbit polyclonal AB2893 Abcam); anti-PARP-1, rabbit polyclonal SC-7150 Santa Cruz Biotechnology); anti-LC3 rabbit polyclonal antibody (NB100-2220, Novus Biological); anti-p62/SQSTM1, guinea pig polyclonal (GP62-C, Progen Biotechnik); anti-actin, goat polyclonal (A1616, Santa Cruz Biotechnology); anti-GAPDH, mouse monoclonal (MAB374, Millipore).

### Immunofluorescence

Human fibroblasts grown on glass coverslips were fixed with absolute methanol at −20°C for 7 min or with 4% paraformaldehyde in 1% PBS at RT for 10 min followed by 0.15% TRITON-X permeabilization. After saturation of non-specific binding with PBS containing 4% BSA, the coverlips were incubated with primary antibodies overnight at 4°C, and revealed with FITC or TRIC-conjugated secondary antibodies (1 hr at RT). Samples were mounted with an anti-fading reagent (Molecular Probes Life Technologies) and observed with a Nikon E 600 epifluorescence microscope. The images captured with NIS-Elements program 4.3, were subsequently modified with Photoshop CS program.

### Western blotting

Cells were lysed in a buffer containing 20 mM Tris-HCl, pH 7.5, 1% SDS, 1 mM Na3VO4, 1 mM PMSF, 5% β-mercaptoethanol and protease inhibitors. Total lysates were diluted in Laemmli buffer, subjected to SDS-PAGE (8% or 4–20% gradient gel) and transferred to nitrocellulose membranes (BioRad) o/n at 4°C. Membranes were saturated with 4% BSA and incubated with primary antibodies for 1 h or o/n. Secondary antibodies were used at 1:15000 dilution for 20 min. Immunoblotted bands were revealed by the Amersham ECL detection system. Intensity measurement was performed using a BioRad densitometer (GS 800) equipped with Quantity One Software.

### Gene Expression Analyses

All samples at the end of the 28 days of treatment were harvested and washed in PBS twice while maintained in ice. Cell pellets were lysed by the addition of 0.5 ml of TRIzol reagent (Invitrogen). RNA was recovered by precipitation with isopropyl alcohol and then treated with DNase I (DNAfree Kit, Ambion). Total RNA was reverse transcribed using the High Capacity RNA-to-cDNA Kit (Applied Biosystems), according to the manufacturer's protocol. Complementary DNA was synthesized from 1 μg of total RNA per sample and analyzed by RT-qPCR. mRNAs of interest were quantified with specific primers: (progerin Fw: 5′-ACTGCAGCAGCTCGGGG-3′, progerin Rev: 5′-TCTGGGGGCTCTGGGC-3′, prelamin A Fw: 5′-ACTGGGGAAGAAGTGGCCAT-3′, prelamin A Rev: 5′-GCTGCAGTGGGAGCCGT-3′, and housekeeping gene glyceraldehyde-3-phosphate dehydrogenase (GAPDH): Fw: 5′-TTGCCCTCAACGACCACTTTG-3′, Rev: 5-CACCCTGTTGCTGTAGCCAAATTC-3. ΔΔCt method was used to quantify relative gene expression levels. Data are representative of three independent biological replicates.

### Electron microscopy

Cell pellets from control and HGPS fibroblasts were fixed with 2.5% glutaraldehyde-0.1 M cacodylate buffer pH 7.3 for 1 h at room temperature. After post-fixation with 1% osmium tetroxide (OsO4) in sodium cacodylate buffer 0.1M for 1 h, the pellets were dehydrated in an ethanol series, infiltrated with propylene oxide and embedded in Epon resin. Ultrathin sections (60 nm thick) were stained with uranyl acetate and lead citrate (10 min each) and were observed at 0° tilt angle with a JEOL JEM-1011 transmission electron microscope, operated at 100 kV. At least 300 nuclei per sample were observed.

### Cytofluorimetry

We performed cell cycle analysis by Muse^®^ Cell Analyzer (Millipore). The cells were removed from the flask with trypsin 1% - PBS 1X, washed 3 times with PBS and resuspended in cold methanol vortexing to avoid cell aggregates. Cell suspension was maintained at 4°C over night up to the analysis. The staining protocol used is conforming to The Muse™ Cell Cycle Assay Kit data-sheet (MCH100106, Millipore).

### Population doubling

Cell proliferation was evaluated using AlamarBlue Assay (BUF012B, AbD Serotec). HGPS cells were seeded at a density of 3 × 10^4^ cells per well in a 24-well plate in minimum essential medium in the presence or absence of treatments. After 12, 24 and 48 hrs the plate was incubated at 37°C and 5% CO2 in humidified atmosphere with completed DMEM containing 10% Alamar Blue solution. After 4 hrs, 200 μl aliquots of cell supernatant were transferred in a 96-well plate and measured. Treated samples, controls and blanks were scanned at 600nm and 570 nm wavelength using a TECAN Infinite^®^ 200 PRO series spectrophotometer.

The percentage of Alamar Blue reduction were calculated according to manufacturer's instructions.

### Statistical analysis

Data are expressed as means ± standard deviation (SD). Statistical analyses were performed using GraphPad Prism 6 program. Comparison between groups was made by one-way ANOVA test. Differences were considered significant at *P* ≤ 0.05 and highly significant at *P* ≤ 0.01. A single symbol (such as *) indicates *P* ≤ 0.05 and a double symbol (**) indicates *P* ≤ 0.01. Statistical significance for RT-qPCR was measured by Student's *t*-test. Differences were considered significant when *p* values were less than 0.05 (indicated by ** for *p* < 0.01 and by *** for *p*-value < 0.001).

## CONCLUSIONS

We deduce that the higher lamin A to progerin ratio we obtain by ATRA plus rapamycin treatment of HGPS fibroblasts is sufficient to repair a series of nuclear parameters resulting in an almost complete nuclear normalization. This result also suggests that the lower level of lamin A resulting from the ATRA treatment is still fully compatible with cellular life and that the decrease in progerin amount is sufficient to prevent general toxic effects, suggesting that there might be a threshold level for progerin toxicity. These observations imply that *in vivo* confirmation of the efficacy of combined ATRA and rapamycin treatment will require a heterozygous animal model of progeria expressing both progerin and wild-type lamin A.

Some hypotheses may be suggested about the mechanism by which ATRA and rapamycin, either singularly or in combination, influence the HGPS cellular phenotype. Upon ATRA treatment, we detect a decrease in mRNA level of both lamin A and progerin with respect to untreated cells suggesting that ATRA is effective on *LMNA* gene at the transcriptional level in HGPS fibroblasts. In addition we demonstrate a definite activation of autophagy which contributes to protein downregulation following ATRA administration. Accordingly, ATRA is known to promote autophagy [34], a mechanism involved in progerin degradation, and also triggered by rapamycin in a selective manner against progerin [24, 25, 36]. So we deduce that both transcriptional and autophagic mechanisms are activated upon treating HGPS cells with ATRA and rapamycin, resulting in the decrease of lamin A, prelamin A and progerin.

In addition, during combined ATRA-rapamycin treatment, further effects may be elicited, resulting from multiple interactions, influencing each other and forming feedbacks. As a final result, a restored chromatin status is reached in HGPS nuclei, with distinct euchromatin and heterochromatin domains. In this status chromatin appears functionally active and more accessible to the nuclear enzymatic machinery, therefore a more efficient DNA repair is made possible [51].

We hypothesize a series of potential advantages in using ATRA-rapamycin treatment in comparison with drugs which target the farnesylation step in the maturation of prelamin/progerin. Farnesylation inhibition causes effectively a reduction of farnesylated progerin (and farnesylated prelamin A), but as major drawbacks, it causes an increase in the amount of non-farnesylated prelamin A and a decrease in the availability of mature lamin A [52].

Furthermore, the decrease in prelamin A we obtain, would suggest testing ATRA efficacy also in prelamin A -accumulating laminopathies other than HGPS, where major nuclear alterations are caused by prelamin A forms [53].

Considering the wide availability of both drugs, which have been in use for years and have been thoroughly tested with regard to dosage, tolerance, side effects and toxicity, their low cost and, most importantly, the severity of HGPS, it appears reasonable to promote ATRA and ATRA-rapamycin clinical testing.

## SUPPLEMENTARY MATERIAL FIGURES


