# Supplementary material for: All-trans retinoic acid and rapamycin normalize Hutchinson Gilford progeria fibroblast phenotype
Source: Oncotarget. 2015 Aug 13;6(30):29914–28. doi: 10.18632/oncotarget.4939 (PMC4745772; doi:10.18632/oncotarget.4939)
Supplement: Supplementary file 1 [file oncotarget-06-29914-s001.pdf]

SUPPLEMENTARY FIGURES

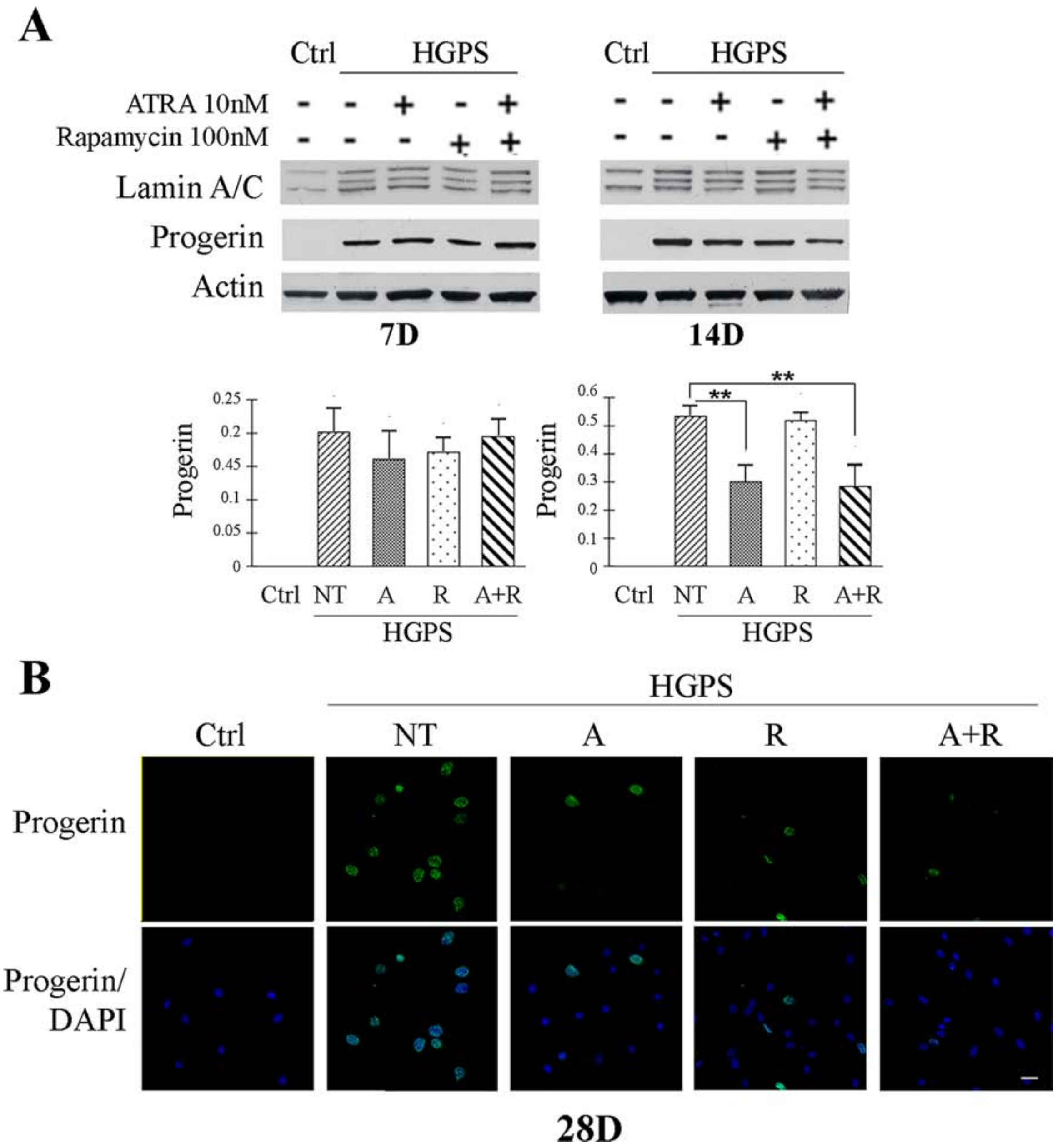

Supplementary Figure S1: A. Treatment effect at different time points. Western blotting analysis of progerin and lamin A/C and relative densitometric quantification at 7 and 14 days of treatment. Actin is used as loading control. Data are presented as means ± SD ($n = 3$); $P = 0.01$(**). **B.** Low magnification figure 1B. Immunofluorescence anlaysis of progerin (green) at 28 days of treatment. DAPI is used as nuclear staining. Scale bar, 10 μM. Ctrl: Healthy fibroblasts; NT: not treated HGPS fibroblasts; A, R and A+R: HGPS fibroblasts treated with ATRA, rapamycin and combined ATRA + rapamycin respectively.

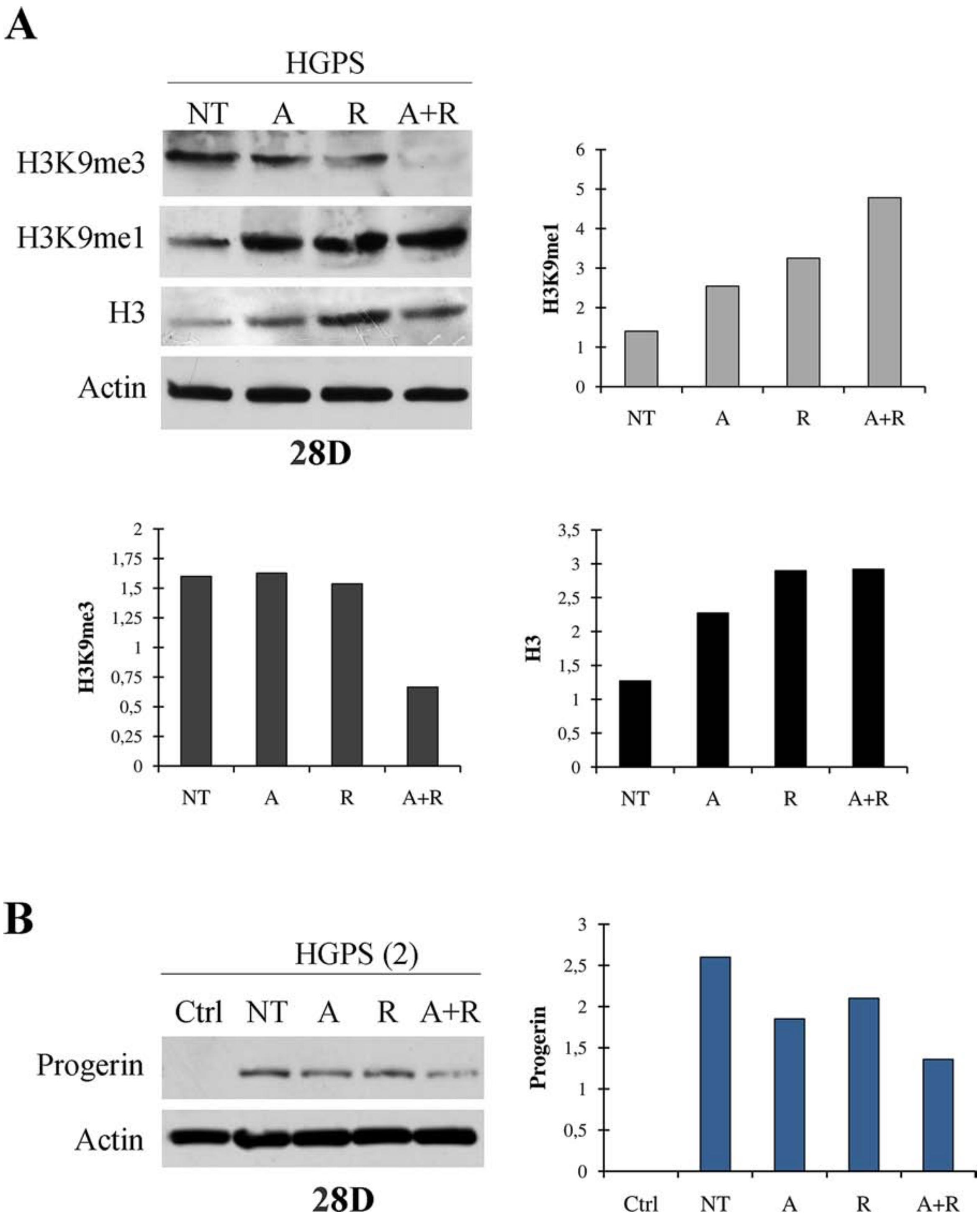

Supplementary Figure S2: A. Western blot analysis of H3K9 (tri-methylated and mono-methylated) and total H3, in untreated (NT) and treated (28 days) HGPS nuclei. A, R and A+R: HGPS fibroblasts treated with ATRA, rapamycin and combined ATRA + rapamycin respectively. **B.** Western blotting analysis of progerin and lamin A/C replicated in cultured fibroblasts from a second HGPS patient.
